# New-Onset Wrist Drop After a Night of Drinking: A Case Report

**DOI:** 10.7759/cureus.58990

**Published:** 2024-04-25

**Authors:** Moronkeji Fagbemi, Narges Joshaghani

**Affiliations:** 1 Internal Medicine and Addiction Medicine, BronxCare Health System, New York, USA; 2 Addiction Psychiatry, BronxCare Health System, New York, USA

**Keywords:** saturday night palsy, compressive radial mononeuropathy, wrist drop, naltrexone, alcohol use disorder

## Abstract

This case report highlights the clinical approach to evaluating a patient with substance use disorder presenting with a sudden onset of peripheral neuropathy in the left hand. Our patient had significant cardiovascular risk factors, which further broadened the differential diagnosis beyond common causes of mononeuropathy. The use of detailed and appropriate clinical history, physical examination, and careful selection of relevant laboratory and radiological tests was instrumental in ruling out multiple medical differential diagnoses, including common mononeuropathies and life-threatening ones, such as cerebrovascular accidents, which facilitated the involvement of necessary consults while also treating both the presenting medical complication and underlying severe alcohol use disorder with additional efforts at relapse prevention.

## Introduction

Alcohol use disorder (AUD) is more common than admitted and often goes undiagnosed. Studies and experience have shown that problem drinkers tend not to seek help until they have advanced dependence, often with associated medical and sociological complications [1]. Excessive alcohol use accounts for 1,714,757 emergency visits yearly as a primary reason in the United States, and harms related to both acute and chronic effects of alcohol contribute to about 5 million emergency department visits and about 178,000 deaths in the US each year [2,3]. Among patients with chronic AUD, neuropathy is the most common harmful sequelae. It is estimated that in the United States, 25% to 66% of chronic alcohol users experience some form of neuropathy [4]. Wrist drop is a disorder caused by radial nerve palsy and those with this problem cannot actively extend the wrist because the radial nerve innervates the extensor muscles of the wrist and digits. A typical scenario elicited in history is when the patient drinks a large amount of alcohol at a party, becomes intoxicated, and perhaps sleeps with his or her body weight on the left arm [5].

The evaluation and diagnosis of compressive radial mononeuropathy are mainly clinical, and patients with a clear history and physical exam do not require further workup. Nevertheless, additional diagnostic tools can help evaluate alternative causes and complications and predict prognosis [6].

This case report analyzes the importance of excellent bedside clinical expertise of good history taking and the use of specific medical maneuvers during detailed physical examination coupled with appropriate tests to help arrive at the diagnosis in a patient with severe AUD presenting with a sudden onset of peripheral mononeuropathy.

The written consent was obtained from the patient to report their case and use their picture in the medical literature.

## Case presentation

A 58-year-old man with a history of hypertension, dyslipidemia, and severe AUD was admitted for alcohol withdrawal and new-onset weakness of the left hand and wrist. He presented with the concern, “Am I having a stroke?” The patient denied any fall, recent trauma to the hand, or weakness of any other part of the body. He had been drinking alcohol most of the night “over a pint of liquor and a few beers” before falling asleep in his dining room. His adult son woke him up from his overnight sleep with his left arm hanging over the dining chair. The patient admitted to some tingling sensation in the left hand and an inability to grasp things well with it, which prompted their visit to the emergency room. The patient denied a history of diabetes and his medication list consisted of amlodipine 10 mg, hydrochlorothiazide 25 mg, and atorvastatin 40 mg once daily.

**Figure 1:**
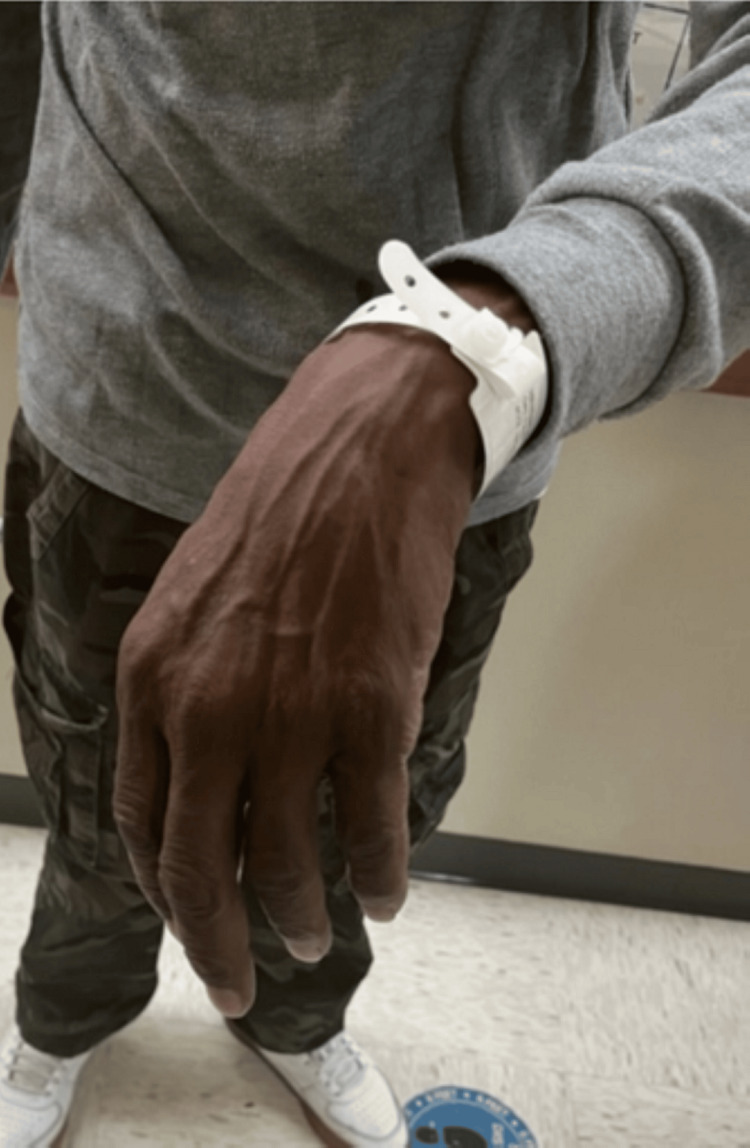
Wrist drop from radial nerve palsy. Picture courtesy of Dr. M. Fagbemi, BronxCare Hospital, Bronx, NY.

The physical examination was unremarkable, except for sinus tachycardia, which was 108/minutes. No focal neurological deficits were noted, except the left wrist drop (Figure 1) and some paresthesia in the posterior aspect of the forearm and the radial aspect of the dorsal left hand and digits.

The Tinel’s and Wartenberg's signs were negative and the patient was unable to perform the hitchhike sign. His Spurling test was also negative. His Clinical Institute Withdrawal Assessment for Alcohol Scale (CIWA-Ar) score was 12.

Laboratory tests were unremarkable, except for urine toxicology positive for ethanol (84 mg/dl) and an electrocardiogram showed sinus tachycardia with a ventricular rate of 102/minute. The computerized tomography of the head was normal.

The patient was admitted into a medically supervised inpatient detoxification with alcohol withdrawal syndrome and radial nerve palsy and initiated on symptom-triggered oral chlordiazepoxide detox protocol. Neurology and physical therapy consults were requested. The patient improved clinically with the improvement of CIWA-Ar score over five days of detoxification. He had daily physical therapy on his left wrist and hand, and also had it placed in a removable splint in a mild extension position. His tingling, paresthesia, and power improved gradually as the patient transitioned from inpatient detox to the inpatient substance use rehabilitation unit. The patient was also placed on pharmacologic treatment with oral naltrexone 50 mg daily to help with abstinence and relapse prevention. The power of the left hand improved to 3 over 5 from an initial score of 1 over 5, and paresthesia and tingling sensations improved significantly. He was directed to follow up at the neurology and outpatient physical therapy clinics post discharge.

## Discussion

The causes of peripheral mononeuropathy in adults range from systemic diseases, such as diabetes, vitamin B-12 deficiency, hypothyroidism, systemic lupus erythematosus (SLE), human immunodeficiency virus (HIV) infection, and syphilis, to local entrapment syndromes. All these differential diagnoses were ruled out in our patient by specific medical maneuvers during physical examination and through pertinent lab results.

Other differential diagnoses include alcoholic neuropathy, cerebrovascular accident, carpal tunnel syndrome, and cervical radiculopathy. The clinical features of alcoholic peripheral neuropathy develop slowly and progress gradually over a period of months and include sensory, motor, autonomic, and gait functioning abnormalities. Painful sensation with or without burning quality is characteristic of the initial and major symptom of alcoholic neuropathy [7]. Progressively, the sensory and motor symptoms and signs extend proximally into the arms and legs, and finally, the gait may become impaired [7]. Even though the patient has risk factors for cerebrovascular accident; unlike his presentation, which was confined to a sudden onset of wrist-drop, acute stroke is usually accompanied by several generalized features. Common presenting symptoms include vertigo and dizziness, altered level of consciousness, paraesthesia and numbness, monoplegia, speech dysfunction, limb ataxia, headache, and visual disturbances [8]. Carpal tunnel syndrome caused by the median nerve compression producing pain and paresthesia in the distribution of the median nerve can be diagnosed by Tinel's sign and Phalen's test [9].

Cervical radiculopathy is diagnosed by Spurling test and positioning the patient to isolate individual reflex arcs is key [10]. Table 1 includes the descriptions of some provocative tests and signs that can help clinicians diagnose upper extremities neuropathies following nerve injury, including Phalen's test for carpal tunnel syndrome in which you ask patients to place their wrist in complete and forced-flexion with dorsal surfaces of both hands opposed for about 60 seconds [11], Tinel's test to diagnose carpal tunnel syndrome by tapping with a finger along the affected nerve [12], Spurling test to diagnose cervical radiculopathy by passive cervical extension with rotation to the affected side and axial compression [13], Wartenberg's sign for the diagnosis of ulnar nerve palsy characterized by an inability to perform adducted digital extension due to weakness in ulnar innervated intrinsic muscles [14], and all of these were absent in our patient. Thumb extension is tested by asking the patient to do a "hitchhiker" sign, which involves flexion of all fingers and hyperextension of the thumb [15], which was positive in this patient.

**Table 1:** Medical tests and maneuvers for neuropathy. The table was created by Dr. Fagbemi.

Test	Description	Diagnostic use
Phalen's test	Ask the patient to place their wrist in complete and forced flexion with the dorsal surfaces of both hands opposed for about 60 seconds. A positive test shows numbness, burning, or tingling over the thumb, index, middle, and ring fingers [11].	Carpal tunnel syndrome with median nerve compression
Tinel’s test	This involves tapping with a finger along the affected nerve causing numbness, tingling, burning sensation, or pain in your wrist and hand [11,12].	Carpal tunnel syndrome
Spurling test	There is a passive cervical extension with rotation to the affected side and axial compression. In a positive test, pain radiates to the shoulder or upper extremity ipsilateral to the direction of head rotation [13].	Cervical radiculopathy
Wartenberg's sign	There is an inability to perform adducted digital extension due to weakness in ulnar innervated intrinsic muscles, and the unopposed action of the slightly medially attached radially innervated extensor digiti minimi resulting in extension and abduction of the 5th digit [14].	Ulnar nerve palsy
Hitchhike sign	This involves the extension of the thumb [15].	Radial nerve palsy

Treatment for compressive radial mononeuropathy is primarily focused on physical rehabilitation with using of a soft wrist dynamic splint that holds the wrist in extension with a full passive range of motion during rehabilitation. Recovery is not rapid, with mild cases resolving at the earliest in two to four months and often much longer [6].

Repeated episodes of compressive neuropathy secondary to excessive alcohol use can worsen the neuropathic damage and prevent full recovery of function, necessitating the importance of AUD treatment. Naltrexone is an opiate antagonist that modifies the hypothalamic-pituitary-adrenal axis to suppress ethanol consumption and help in alcohol abstinence and relapse prevention [16].

## Conclusions

Excessive alcohol use accounts for a significant number of emergency visits yearly as a primary reason, and even more for alcohol-related visits with varied indirect complications, including compressive neuropathy, as in the case discussed. This can at times mimic other pathologies, hence requiring careful detailed medical history, physical examination, and selected laboratory and radiographic tests when necessary to rule out pertinent differential diagnoses, while also comprehensively treating both the medical complication and underlying AUD, including strategies for alcohol relapse prevention.
